# Minding the gap: discovering the phenomenon of chemical transmission in the nervous system

**DOI:** 10.1007/s40656-023-00591-6

**Published:** 2023-10-25

**Authors:** William Bechtel

**Affiliations:** Department of Philosophy, University of California, San Diego; La Jolla, CA 92093-0119 USA

**Keywords:** Phenomena, Scientific discovery, Electrical transmission, Chemical transmitters, Neurotransmitters

## Abstract

The neuron doctrine, according to which nerves consist of discontinuous neurons, presented investigators with the challenge of determining what activities occurred between them or between them and muscles. One group of researchers, dubbed the sparks, viewed the electrical current in one neuron as inducing a current in the next neuron or in muscles. For them there was no gap between the activities of neurons or neurons and muscles that required filling with a new type of activity. A competing group, the soups, came to argue for chemicals, subsequently referred to neurotransmitters, as carrying out the activities between neurons or between neurons and muscles. But even for them the conclusion that chemicals performed this activity was only arrived over time. I examine the prolonged period in which proponents of chemical transmission developed their account and challenged the sparks. My goal is to illuminate the epistemic processes that led to the discovery of a new scientific phenomenon—chemical transmission between neurons.

## Introduction

Much of the philosophical literature on scientific discovery has focused on the discovery of mechanistic explanations, explanations that appeal to the components of a mechanism to reveal how it generates a phenomenon (Darden, 1991, 2006; Bechtel & Richardson, 1993/2010; Bechtel, 2006; Craver & Darden, 2013). This makes sense because, in discovering mechanisms, search strategies are used to identify components, determine what activities they perform, and how they are organized. But what about phenomena themselves? Following the pioneering contributions by Hacking (1983) on how phenomena are constituted in laboratories and by Bogen and Woodward (1988) showing that explanations are directed at phenomena, not data, philosophers analyzing the practice of mechanistic explanation have emphasized the central role characterizations of phenomena plays in mechanistic explanations (Glennan, 1996, 2017).^1^ There is now growing interest by philosophers of science in how scientists investigate and reason about phenomena themselves, but less discussion of how scientists discover new phenomena. This leaves the question: Are search strategies involved in the discovery of new phenomena?

Much of the philosophical discussion of phenomena has focused on how accounts of them are revised. Without addressing how new phenomena are identified, Bechtel and Richardson (1993/2010) described how inquiry into the responsible mechanism can lead researchers to *reconstitute* the phenomenon they are seeking to explain. Subsequently, several philosophers have argued that reconstitution or revision of the characterization of phenomena often results from investigations directed at the phenomenon, not the mechanism taken to be responsible for it. Keeping the language of reconstitution, Bollhagen (2021) describes how, in the course of developing a research tool, the single-molecule motility assay, to study the phenomenon of the molecular motor kinesin walking hand-over-hand along microtubules, researchers encountered an experimental dead-space. To enter this space, researchers identified a different criterion for characterizing the phenomenon, torque generation and, using it, discovered that kinesins engaged in an unexpected form of hand-over hand progression—asymmetric hand-over-hand movement. Focusing on the phenomenon of memory transfer between organisms that many investigators in the mid-20th century took to be real, Colaço (2018) shows how the characterization scientists offer of a phenomenon is defeasible, and when defeater evidence is advanced, it can lead the research community to dismiss the phenomenon. Turning to the phenomenon of long-term potentiation, Colaço (2020) develops an analysis of the processes through which researchers revise their characterization of a phenomenon they continue to accept as real, showing that conflicting mechanistic evidence typically leads only to a suspension of judgment and subsequent engagement in additional research directed at the phenomenon itself is what leads to a more adequate characterization of the phenomenon.^2^

How are distinctively new phenomena discovered? In some cases, they intrude in the course of ongoing scientific investigations. Investigatory strategies are then invoked to characterize the features of the phenomenon and the variety of conditions under which it can be brought about. Bechtel and Vagnino (2022) analyzed such a case. After discovering that contact between two metals, one touching the muscle and another touching the nerve of a prepared frog, resulted in muscle contractions, Galvani (1791) investigated the circumstances and argued for a new phenomenon he called *animal electricity*. Once tools such as galvanometers were available, other researchers extended this inquiry and further investigated what was happening. This culminated in Bernstein’s (1902) characterization of the membrane potential and electrical transmission along neurons in terms of the propagation of a reduction of the membrane potential. The reduction in the membrane potential was subsequently named the *action potential*. The discovery of the membrane potential and the action potential can be described as starting with an intrusion in an experimental set up that resulted in an extended investigation as to what was happening, culminating in the characterization of two new phenomena.

Here I focus on a different process through which researchers identified a previously unsuspected phenomenon: chemical transmission between neurons. In this case the discovery of a discontinuity between the neurons along which electrical current was transmitted and the associated gap^3^ between the activities of individual neurons, presented the question of what process or activity bridged the gap. Researchers eventually identified a different type of activity, the release and uptake of chemicals, as responsible for bridging the gap. This case differs from both types of cases discussed above. It did not involve the reconstitution, revision of the characterization, or rejection of an existing phenomenon. And the new phenomenon did not intrude into ongoing experiments.^4^ It was identified as researchers discovered that a different type of process mediated between neurons than occurred along their membranes. This discovery was not straightforward and required investigations over several decades. The gap between the activities of neurons that would eventually be mediated by chemical transmission was first noted in the late 19th century. A general consensus that chemical transmission mediated between neurons only developed in the 1950s.

One reason it took such a long time was that many researchers who focused on nerves as engaged in electrical transmission did not recognize that mediation between neurons involved a different type of activity than that which occurred in neurons. They assumed that the electrical current transmitted in one neuron could itself induce electrical current in the recipient neuron or muscle cell. Another community of researchers were investigating the effects of chemicals on various tissues, including on muscles, but initially they downplayed the possibility that these chemicals might figure in mediating transmission between neurons or neurons and muscle. In the 1920s those investigating these chemical effects began to generate findings that suggested a role for chemicals in the transmission between nerves and cardiac or smooth muscles: Otto Loewi devised an experimental strategy in which only chemicals could mediate the transmission between nerves and heart muscle and Henry Dale provided compelling evidence that the suspected chemicals occurred naturally in animals. By the time Loewi and Dale were awarded the Nobel Prize for their contributions to this research in 1936, Dale and his collaborators had begun to argue for a larger role for chemical transmission between neurons. Defenders of the default electrical-transmission account, however, articulated and defended it as still the most compelling account of transmission at junctions between neurons and skeletal muscles and at synapses in the central nervous system.

This often acrimonious debate between the proponents of chemical transmission and those advocating electrical transmission came to be known as the war between the soups and the sparks.^5^ Over the 15 years after Loewi and Dale were awarded the Nobel Prize, researchers favoring the chemical hypothesis gradually accumulated evidence that the gap between activities within neurons was mediated in all cases by chemical transmission. The sparks, however, were not persuaded by such evidence and continued to find their default assumption of electrical transmission compelling. The break came when Eccles, who had been the leading champion of the electrical hypothesis, formulated it in a sufficiently precise form to test it experimentally. The results Brock et al. (1952) generated were the opposite of what Eccles had predicted from his account of electrical transmission. Unable to generate a competing precise formulation of the electrical hypothesis that was compatible with his experimental results, he switched his allegiances. In the following years a new research program developed as researchers began to identify different chemical transmitters and characterize their effects.^6^

For theorists seeking to understand how scientists make discoveries, cases in which discovery required an extended period of time provide opportunities for identifying the steps through which discovery is made. Rheinberger (1997), for example, took advantage of such an extended process to reveal how experimental intrusions became what he referred to as epistemic things in the course of developing explanations of protein synthesis. In this paper I take advantage of this protracted process to examine how the soups and sparks respectively reasoned about the gap between the activities within neurons and interpreted experiments suggesting what mediated it. In this case members of both groups often participated in the same conferences and published in the same journals—notably, *The Journal of Physiology* and *The American Journal of Physiology*. However, their reasoning and experimental approaches reflected their different disciplinary backgrounds. The proponents of the electrical hypothesis were the descendants of the 19th century researchers who investigated the transmission of electrical pulses along nerves before the gap between neurons was identified. The 19th century researchers relied on the galvanometer to measure current and rheotomes to control the location and timing of stimulation and detection of a response (Hoff & Geddes, 1957). In the first half of the 20th century researchers in Britain (Charles Sherrington, Edgar Douglas Adrian; for discussion, see Bennett, 2001) and in the United States (Hallowell Davis, George Bishop, Detlev Bronk, Joseph Erlanger, John Fulton, Herbert Gasser, Ralph Gerard, and Rafael Lorente de Nó^7^) continued this project of recording and characterizing the electrical activity in nerves and eventually in individual neurons.^8^ Given their tools, these researchers embraced the default assumption that the same electrical activity they detected in neurons continued across the synapse.

The proponents of the chemical hypothesis, on the other hand, had roots in investigations testing the effects of chemicals, either extracted from plants or animals or synthesized in laboratories, on living organisms. Plant substances that produced effects on animals were referred to as *Materia medica* and university researchers who investigated them often held positions of Professor of Materia Medica. As described by Valenstein (2005, p. 7), these practitioners developed a variety of physiological responses to assay the effects of drugs as they investigated “how drugs exerted their effects and what they could reveal about normal physiology.” They investigated, among other effects, those of drugs on blood pressure, heart rate, salivation, glandular secretions, vasoconstriction and dilation, and contraction and relaxation of both smooth and skeletal muscles. Eventually, these endeavors gave rise to the experimental discipline of pharmacology. Even so, for a prolonged period most of these researchers resisted the proposal, advanced by some of them, that these chemicals provided the vehicle for transmission between neurons and muscles and even between neurons themselves.

Much of the research by both soups and sparks consisted in generating specific findings—developing experimental interventions and procuring data about what happened in response. Both soups and sparks produced a wealth of such findings, resulting in voluminous publications. But, as Bogen and Woodward (1988), in their pioneering work on phenomena, emphasize, phenomena are not data. Phenomena are regular occurrences taken to occur in the world, data are specific findings that provide evidence for them.^9^ The transition from procuring data to identifying regular occurrences in the world requires deploying conceptual tools that allow researchers to characterize what they take to exist (Feest, 2010, 2011; Haueis, 2023). While embracing their conclusions, my focus is on how the recognition of a discontinuity between neurons influenced the identification of a new phenomenon that needed to be characterized conceptually. I discuss how proponents of both the electrical and chemical hypotheses developed their conceptualizations of the types of processes they took to be occurring at synapses and employed these in understanding their experimental results.

Although research relevant to both the electrical and the chemical hypotheses was already developing, the recognition that there was a gap between activities that required mediation only arose with the acceptance at the end of the 19th century of Ramón y Cajal’s claim that nerves consist of discrete neurons and Sherrington’s naming the point of contact between discrete neurons the *synapse*. This is the focus of Sect. 2. In Sect. 3 I examine how proponents of the electrical hypothesis denied the gap between processes within neurons, viewing the processes occurring at the synapse as a continuation of those occurring in neurons and muscle cells. In Sects. 4, 5, and 6 I investigate the development of the chemical hypothesis, beginning in Sect. 4 with early proponents whose articulation of the proposal had little uptake. Section 5 examines Dale’s reasons for not embracing the chemical hypothesis in 1914 while Sect. 6 turns to the contributions of Loewi and Dale in the 1920s in establishing the role of the release and uptake of acetylcholine at neuromuscular junctions in the autonomic nervous system. Already before they received the Nobel Prize for this work in 1936, Dale and a cadre of researchers began to push the chemical hypothesis beyond the autonomic neuromuscular junction to the neuromuscular junction with striate muscles and at synapses between neurons in the central nervous system. This effort is the focus of Sect. 7. Section 8 examines how the widespread recognition that the chemical hypothesis could explain the autonomic neuromuscular junction galvanized the opposition of proponents of the electrical hypothesis, especially Eccles, to articulate arguments against the chemical hypothesis. As discussed in Sect. 9, after Eccles formulated a proposal for how electrical signaling could be inhibitory, he generated evidence that strongly supported the chemical hypothesis. After he switched his allegiance, the controversy petered out and most researchers embraced the chemical hypothesis. I conclude in Sect. 10 by drawing together the various insights this case provides about the process of establishing a new activity occurring in the gap between the entities involved in existing phenomena and thereby a new scientific phenomenon.

## Revealing a gap

Prior to the recognition of neurons as distinct cells, researchers did not recognize a gap in the activity in nerves that needed to be bridged for nerves to transmit electrical activity. Instead, investigators who focused on nerves treated them as continuous structures. Early studies of electrical transmission in animals focused on nerves but even more on muscle. At the end of the 19th century Galvani (1791) defended the existence of a source of electricity in muscles; to make sense of this finding, he drew an analogy between muscles and Leyden jars. Galvani could only detect this current with a prepared frog muscle, but the introduction of the galvanometer in the 1820s spurred detailed investigations of current in muscle and nerve. du Bois-Reymond (1852) not only characterized these currents in both muscle and nerve, but also argued that the signal transmitted along nerves or muscles was in fact a reduction of the currents generated in nerve or muscle. One of his students, Hermann (1870; Burdon-Sanderson, 1878), showed that current only arose when nerve or muscle was injured (for which he was expelled from du Bois-Reymond’s laboratory), while another, Bernstein (1902, 1910), advanced and defended the hypothesis that what exists in uninjured muscle and nerve is a membrane potential due to ion gradients across the membrane. The reduction in the potential, subsequently referred to as the *action potential*, is what is transmitted along nerves.

This research took for granted that an electrical signal conducted along a nerve could be transmitted to muscle without inquiring into how. A passage from a relatively late two-volume work of du Bois-Reymond’s is often cited as setting the stage for the debate over whether such transmission is electrical or chemical: “Of known natural processes that might pass on excitation, only two are, in my opinion, worth talking about: either there exists at the boundary of the contractile substance a stimulatory secretion in the form of a thin layer of ammonia, lactic acid, or some other powerful stimulatory substance; or the phenomenon is electrical in nature” (du Bois-Reymond, 1877, p. 700; translation by Bacq, 1975, p. 11). Du Bois-Reymond’s discussion is invoked by proponents of both sides as supporting their alternative. Krnjević (1974), however, argues that Bernard was actually addressing a different issue: he assumed that the nerve terminal penetrated the muscle membrane and was discussing the intracellular communication between the endplate and contractile substance of the muscle. According to Krnjević, Bernard assumed that if the nerve remained outside the sarcolemma, “the mechanism of neuromuscular transmission could only be electrical.”

The recognition that animal tissue consisted of distinct cells arose from a different line of research that employed microscopes to observe biological tissues. By the 1830s designers of microscopes had succeeded in reducing the most serious aberrations produced by earlier microscopes. The improved microscopes sufficed to enable researchers to observe cells; Schwann (1839/1947) is widely credited with establishing that animal tissues consist of discrete cells.^10^ In the middle of the century researchers developed techniques for applying stains to enhance the contrast between cells and their background. In the case of nerve cells, stains revealed the axons and dendrites protruding from them. Among the various stains introduced, the silver nitrate stain introduced by Golgi (1883) proved especially effective in revealing neurons. Since it only stained a small percentage of neurons in the tissue being observed, the silver nitrate stain made the distinctness of individual neurons easier to recognize. Golgi, however, rejected the claim that neurons were in fact distinct. Instead, he interpreted his observations as revealing that dendrites formed a reticular network running throughout the nervous system. Ramón y Cajal (1894/1990) challenged this interpretation, arguing that stained neural tissue revealed neurons as distinct cells. He could not, however, see a space between neurons; he inferred that they were distinct from each other from such evidence as that, in developing tissue, dendrites and axons grow and that when the axon is severed from the cell body, the resulting atrophy terminates at the boundary of the next cell.

Sherrington, in collaborating with Foster on the third part of his *Textbook of Physiology*,^11^ drew attention to the discontinuity between neurons and proposed the name *synapsis* (to clasp) for what he referred to as the “special connection” between them:

So far as our present knowledge goes we are led to think that the tip of a twig of the arborescence [from an axon] is not continuous with but merely in contact with the substance of the dendrite or cell-body on which it impinges. Such a special connection of one nerve-cell with another might be called a *synapsis.* (Foster, 1897, p. 929)

Just after introducing the term *synapsis* Foster/Sherrington continues to consider how, despite the discontinuity, the processes or activities in one neuron induce those in the next: “it has been suggested that the lack of continuity between the material of the arborisation of the one cell and that of the dendrite (or body) of the other cell offers an opportunity for some change in the nature of the nervous influence as it passes from the one cell to the other. But this must be regarded at present as a useful suggestion rather than as a definitely proved truth” (p 930).

With the widespread acceptance of the neuron doctrine and Sherrington’s characterization of the synapse, nerves ceased to be viewed as continuous tissues but rather as comprising separate cells with a gap between the processes in one and those in the next. This posed the question: how are electrical currents within neurons communicated between neurons and between neurons and muscles?^12^

## Denying the significance of the gap: electrical transmission as the default position

Many investigators assumed that despite the distinct identity of neurons, the electrical current in one neuron would induce the current in the next. The simplest assumption was that the material filling the gap would transmit electrical currents just as the membrane of the neuron did. Lucas (1907), for example, proposed that *junctional materia* filled the space between neurons and transmitted current between nerves. Others, such as Louis and Marcelle Lapicque, proposed that electrical currents could be transmitted across the synapse through alignment of what they termed the *chronaxie* in each neuron. They characterized chronaxie mathematically: the minimum time required for a constant current to double the intensity of the minimum current to which the tissue would respond (Lapicque, 1909; Irnich, 2010). They offered an account according to which chemical agents such as curare altered responses by changing the chronaxie of neurons so that they no longer aligned.

These proposals, however, were the exception. Rather than hypothesizing or defending proposals about how processes at the synapse conducted electrical activity from one neuron to the next or to a muscle cell, most researchers simply carried on with their efforts to record electrical activity, using newly developed intervention tools such as electrodes that could be inserted into ganglia or neuromuscular junctions and measurement devices such as the string galvanometer and the cathode ray oscilloscope. Using such instruments, Lucas (1909) established that when stimulated, muscle fibers respond in an “all-or-none” manner, and Adrian (1914) extended the principle to individual neurons. In the U.S., Gasser and Erlanger (1922) established that action currents (which, after 1924 they referred to as *action potentials*) in nerve trunks exhibited complex wave forms, which they interpreted as reflecting different fibers conducting electrical signals at different velocities. Combining this with information about conduction velocity and fiber diameter, they developed a classification of nerve fibers and the speeds at which they transmitted.

When electrophysiologists did address the chemical hypothesis, their most common objection was that chemical transmission was too slow to account for the transmission between nerves or between nerves and skeletal muscle. The delays in electrical activity crossing synapses were typically shown to be on the order of 1–2 msec. It was generally assumed that chemical transmission would take much longer. After completing his dissertation under Sherrington (on inhibitory processes in the spinal cord), Eccles provided a measurement of how slow chemical transmission might be, using inhibition of the heart by the vagus nerve as a model. By then there was compelling evidence, accepted even by the proponents of the electrical hypothesis, that acetylcholine transmitted the signal from the vagus nerve to the heart. Brown and Eccles (1934) demonstrated a latency of 100–160 msec in the response of the heart, which they inferred was mostly due to the time required for release of acetylcholine. For many this finding was compelling proof that chemical transmission was two orders of magnitude too slow to account for transmission at synapses in the brain or at skeletal neuro-muscular junctions.^13^

## Filling the gap with chemicals: three early proponents (1900–1910)

Independent of research on nerve transmission, practitioners of *Materia Medica* explored the effects of plant compounds on animals and compared their effects to those of nerves. For example, Schmiedeberg and Koppel (1869) extracted muscarine from the mushroom *Amanita muscaria* and demonstrated that it caused slowing of contractions of cardiac muscles. They further demonstrated that these effects could be countered by atropine, purified from the poisonous plant *Atropa belladonna*. Numerous researchers noted that the effects of muscarine were similar to those produced by stimulation of the vagus nerve. This, however, did not lead them or others at the time to infer that a chemical such as muscarine was in fact released by the vagus nerve and acted on muscle.^14^ Physiologists such as Claude Bernard also explored the effects of plant material. Bernard (1849) investigated the poisonous effects of curare, an extract of the plant *Strychnos toxifera* used by the Macusi people of Guyana to coat the tips of arrows. Bernard demonstrated that after administration of curare to a rabbit, electrical stimulation of a nerve would no longer result in muscle contraction. Yet, the muscle itself would still contract if an electrical stimulus was applied directly to it. Since the nerve was not damaged, Bernard inferred that curare acted on the neuro-muscular junction (Bennett, 2001).^15^

These investigations provided the backdrop for three researchers who, in the first decade of the 20th century, argued for chemical transmission between autonomic nerves and muscle. Although all three were respected as credible researchers and published their hypotheses in prominent venues, there was no immediate uptake of their proposals.

John Langley was a distinguished senior investigator whose earlier contributions included differentiating the sympathetic and parasympathetic nervous systems and demonstrating their opposed effects on specific organs. He also followed up on Bernard’s research on the effects of curare. By applying miniscule amounts from a thread dipped in curare, he showed that it only acted at the point where the nerve and muscle met. He also showed that nicotine also only produced effects when applied at the nerve-muscle junction. In some cases, he found that nicotine first caused muscles to contract repeatedly and, only when they were exhausted, inhibited contraction. To explain the ability of chemicals (including curare) to induce contraction, he proposed that the muscle tissue included a “receptive substance” that “receives the stimulus and, by transmitting it, causes contraction” (Langley, 1906, p. 182; for an account of the history of the understanding of drug receptors, see Maehle et al., 2002). From this, Langley advanced an argument that transmission from nerve to muscle was chemical: “the nervous impulse should not pass from nerve to muscle by an electric discharge, but by the secretion of a special substance at the end of the nerve, a theory suggested in the first instance by du Bois-Reymond.” (p. 183).^16^ Among the substances Langley investigated was adrenaline, extracted from the adrenal gland. Adrenaline came to his attention from reports by Oliver and Schäfer (1895) of dramatic rises in blood pressure after its administration.^17^ Langley (1901) described a broader range of effects: slowing intestinal peristalsis, dilating the pupil, and increased salivation. He stressed the parallel between administering the extract and stimulating the sympathetic nervous system and demonstrated that the effects occurred even after the nerves were cut and degenerated.

Thomas Renton Elliott, working as an undergraduate under Langley, compared the effects of adrenaline^18^ with those produced by stimulation of sympathetic nerves and showed that the effects were not reduced after the degeneration of the nerves as a result of lesioning the ganglion cells from which they originated. He concluded his short published abstract (Elliott, 1904, p. xxi) with the proposal “Adrenaline might then be the chemical simulant liberated on each occasion when the impulse arrives at the periphery.” Langley (1905) embraced the proposal and proposed that adrenaline acts on a specific receptor substance: the “action of adrenalin depends upon the presence in the muscle protoplasm of some substance” (p. 375) with which adrenaline combines. He further proposed that “both inhibitory and motor substance[s] might be present [at a synapse, and thus] the effect of a nervous impulse depends upon the proportion of the two kinds of receptive substances” present” (p. 412). Even with Langley’s endorsement, Elliott did not continue to advocate for his proposal. In his longer report of his experiments, Elliott (1905) only mentions it as one of several possibilities “the conjecture that [adrenaline] is concerned in the transference of a sympathetic nervous impulse, and stored to such an end in the neighbourhood of the myoneural junction” and concludes “The evidence does not conclusively disprove any of these.”^19^

The third early advocate of chemical transmission, Walter Dixon, explored the effects of numerous pharmacological agents, including mescal. He was inspired, in part, by Elliott’s results to advance a bold hypothesis “when a muscle contracts, when a gland secretes, or a nerve ending is excited, the cause in each case may be due to the liberation of some chemical substance, not necessarily set free in the circulation as in the case of secretion, but more likely liberated at the spot upon which it is required to act” (Dixon, 1907, p. 454; see also Dixon & Hamill, 1909). To test this idea, Dixon applied a stimulus to the vagus nerve to the heart^20^ for a half hour and made an extract. When he applied the extract to second heart, contraction was inhibited. The effect was blocked by applying atropine. He compared the effect to that of muscarine and offered an explanation:

I interpret these experiments to mean that some inhibitory substance is stored up in that portion of the heart to which we refer as a ‘nerve ending,’ that when the vagus is excited this inhibitory substance is set free, and by combining with a body in the cardiac muscle brings about the inhibition. If cardiac inhibition is brought about in this way, drugs must act by liberating the inhibitory hormone. Atropine either prevents the liberation of the hormone, or saturates the substance in the end organ upon which it acts. The former seems the more probable explanation. (p. 454)

Like Elliott, Dixon did not pursue this hypothesis. In his obituary of Dixon, Gunn (1932, p. 12) relates “I once asked him why he dropped the subject and he said that he was deterred by the universal scepticism with which his views were received.”

The hypothesis that the gap between activities in neurons and in heart muscle might be bridged chemically was clearly developed and defended experimentally in the first decade of the 20th century. It was not refuted, but rather ignored. As there is no record of arguments opposing Langley’s, Elliott’s, and Dixon’s proposals, we can only speculate about why this was the case.^21^ It is not surprising that those investigating the electrical properties of nerve transmission ignored them—they didn’t recognize a gap that needed bridging. It is more difficult to explain why those investigating the effects of various chemicals on organisms didn’t pick up on the hypothesis. Their focus seems to have been more broadly devoted to establishing pharmacological effects of various chemicals, not on explaining physiological processes. There was no evidence that the chemicals that yielded the effect were themselves generated at the neuromuscular junction. Ultimately it was evidence that noradrenaline and acetylcholine occurred naturally in animals that brought Dale to accept that they functioned as transmitters.

## Elaborating on the candidates: Dale’s investigations of adrenaline and acetylcholine

As a result of his studies of a number of chemicals, most notably adrenaline, acetylcholine, and histamine, Dale was the researcher who was best prepared to pursue the chemical hypothesis in the following decade. Despite showing that these chemicals had effects comparable to nerve stimulation, however, he did not then view them as involved when neurons initiated the response. They only simulated what happens between nerves and muscle. The reasons someone as well positioned as Dale held back from adopting the chemical hypothesis are informative of what it would take to establish the phenomenon of chemical transmission.

In 1904, while completing his medical studies, Dale accepted a position as a pharmacologist and, in 1906, as director of research at Wellcome Physiological Research Laboratories. There he was challenged by Henry Wellcome to investigate the pharmacology of ergot, a fungus that grows on rye and related plants. In ancient Assyria it had been used as a poison but had attracted medical interest when it was found by midwives to speed up labor. In the course of these investigations, Dale, together with a chemist, Barger, found adrenaline, acetylcholine, histamine, and several other amines in ergot extracts and began investigating their physiological effects. Barger and Dale (1910) demonstrated that adrenaline as well as dopamine and catecholamine increased the blood pressure of anesthetized or spinal cats. Since the effects were similar to those produced by stimulating the sympathetic nervous system, Barger and Dale referred to them as *sympathomimetics*. They further demonstrated that, of all these substances, noradrenaline produced effects that most closely approximated those of nerve stimulation. His main reason for not interpreting noradrenaline was a chemical mediator between sympathetic neurons and muscle was that, unlike adrenaline, there was no evidence that noradrenaline occurs in animals.

Much of Dale’s research while at Wellcome focused on acetylcholine. Acetylcholine was first identified as a synthetic substance when von Baeyer (1867) produced it by acetylizing choline. von Baeyer was drawn to choline when he showed that *neurin*, which Liebreich (1865) had extract from brain material, was choline. Physiological interest in acetylcholine followed Hunt’s (1900) demonstration that after removing adrenaline from adrenal extract, the remaining extract would cause blood pressure to drop. Hunt proposed this might be due to an ester of choline and, after testing the effectiveness of a number of choline derivatives on reducing blood pressure and heart rate, found that acetylcholine had the greatest effect: “It is one hundred thousand times more active than cholin, and hundreds of times more active than nitro-glycerine; it is a hundred times more active in causing a fall of blood pressure than is adrenalin in causing a rise” (Hunt & de M. Taveau, 1906, p. 1789). In the course of studying a contaminant in his preparations of ergot that acted to suppress contractions of heart muscle and other organs, Dale first hypothesized that it might be muscarine. When he found it was far more labile than muscarine, he tested acetylcholine, which had just be synthesized by Ewins (1914), the chemist with whom he was working. Finding that it produced the same effects as the ergot contaminant, he concluded that the contaminant was acetylcholine and that its appearance in ergot established that it did occur naturally in living organisms.

Dale went on to conduct a thorough study of acetylcholine, finding that in addition to its muscarine-like effects on the heart, which were blocked by administration of atropine, it had effects on skeletal muscles that could not be blocked by atropine. These, though, were inhibited by curare. He introduced the distinction between the muscarinic (slow) and nicotinic (fast) actions of acetylcholine, which much later could be understood in terms of the difference between ionotropic and metabotropic receptors. He also proposed that its lability might be due to an esterase that rapidly degraded it (Dale, 1914). Dale was clearly enticed by the similarities of the effects generated by applying acetylcholine to those generated by excitation of parasympathetic nerves. In 1913 he wrote to Elliott: “We got that thing out of our silly ergot extract. It is acetyl-choline and a most interesting substance. It is much more active than muscarine, though so easily hydrolysed that its action, when it is injected into the blood-stream, is remarkably evanescent, so that it can be given over & over again with exactly similar effects, like adrenaline. Here is a good candidate for the rôle of a hormone related to the rest of the autonomic nervous system. I am perilously near wild theorising” (Letter, Dale to Elliott, December 11, 1913, quoted in Valenstein, p. 44). He even broached the topic in his 1914 paper: “The question of a possible physiological significance, in the resemblance between the action of choline esters and the effects of certain divisions of the involuntary nervous system, is one of great interest, but one for the discussion of which little evidence is available. Acetyl-choline is, of all the substances examined, the one whose action is most suggestive in this direction” (p. 188). But after recounting the grounds for speculation, he concluded “there is no known depôt of choline derivatives, corresponding to the adrenine depôt in the adrenal medulla, nor, indeed, any evidence that a substance resembling acetyl-choline exists in the body at all” (p. 188). At this point, Dale suspended his research on acetylcholine. During World War I, he concentrated on administrative responsibilities. After the war, he turned his focus to histamine and its role in vasodilation (Dale & Laidlaw, 1919).

In the course of his chemical inquiries, Dale had established a number of pharmacological effects of noradrenaline and acetylcholine and showed that they emulated the effects of stimulation by nerves in the parasympathetic and sympathetic nervous systems. He even seems to have been tempted by the hypothesis that they were the agent produced by the nervous system that acted on muscle. But he held back. The reason his gives is that there was no evidence that acetylcholine or noradrenaline occurred in animals. If that were his sole reason, however, one would have imagined that Dale would have prioritized testing for their occurrence in animals. But he did not, and only returned to that inquiry a decade later in the course of trying to establish the origin of histidine in animals. This suggests that he did not find the idea of chemical transmission at neuromuscular junctions to be sufficiently compelling to make it worth pursuing it.

## Loewi and Dale: establishing chemical transmission at the autonomic nerve-muscle junction

**Fig. 1:**
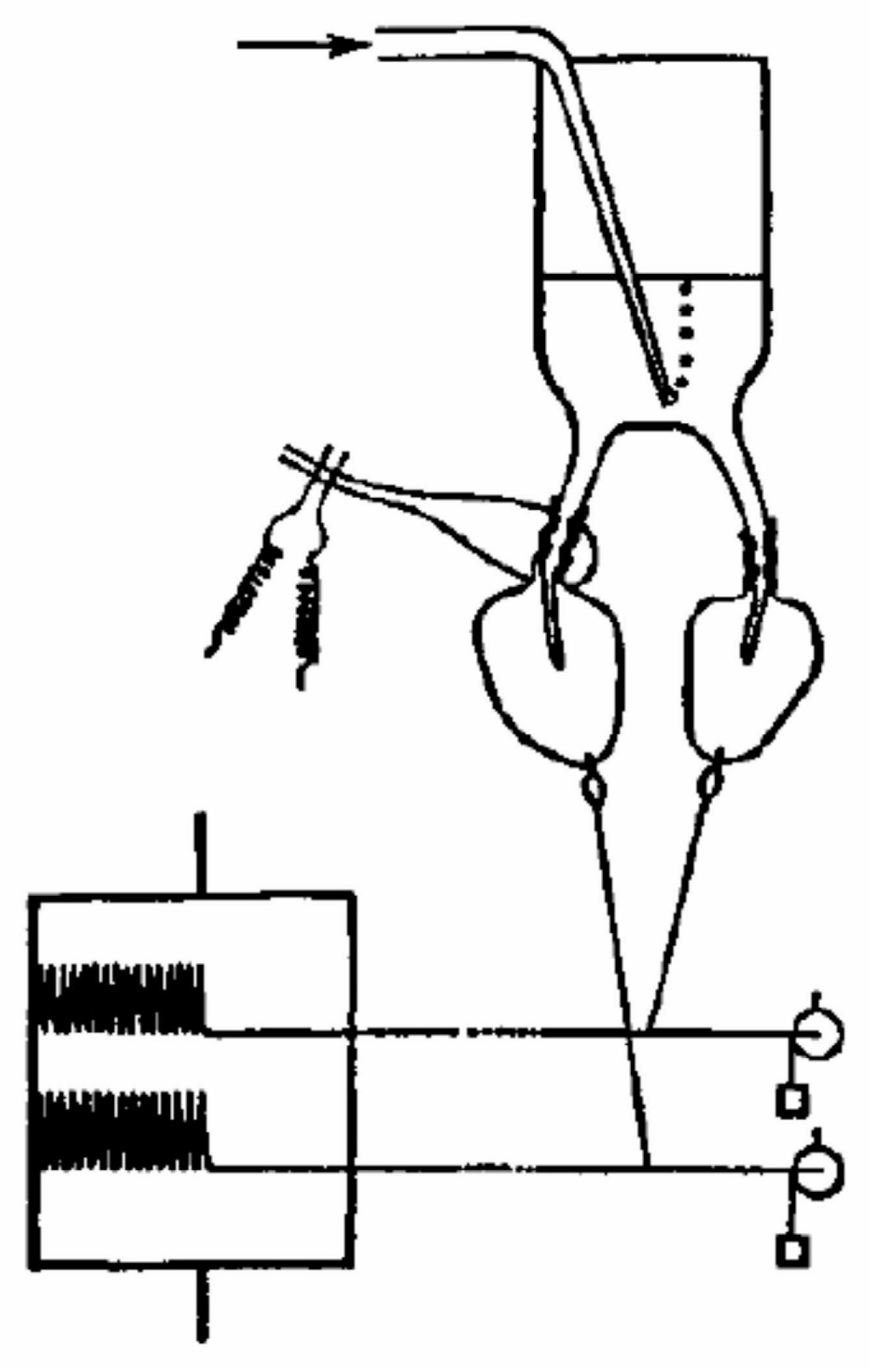
Kahn (1926) version of Loewi’s experiment, using a common cannula for both hearts in which stimulating one heart resulted in stimulation of the other

Of all the research reviewed so far, only that of Dixon had been directed at experimentally investigating whether a chemical compound extracted when a heart was stimulated would produce the same effect on another heart. In 1921 Loewi carried out a conceptually similar experiment to Dixon’s.^22^ He prepared two frog hearts in saline solution and then stimulated the vagus nerve still attached to one of the hearts to depress its activity. He then transferred some of the solution to the other heart, whose vagus nerve had been removed. Its activity was thereby depressed (Loewi, 1921). (Fig. 1 shows a modification of Loewi’s procedure by Kahn, 1926, that avoids the need to transfer the saline solution from one heart to the other.) Since the only way one heart could affect the other was via the saline solution, Loewi concluded that some chemical—he called it *Vagusstuff*—was released by the nerve connected to the first heart and communicated to the other. Loewi followed this experiment with one in which he first stimulated the sympathetic nervous system, accelerating the frog heart. Again, when the solution in which the heart was situated was transferred to another heart, its activity was accelerated. Loewi referred to the chemical which he concluded was released from the sympathetic nervous system as *Acceleransstoff*.^23^

Loewi’s report attracted a great deal of interest, but also considerable criticism. The criticism partly stemmed from the fact that many attempts to replicate his experiment failed, including attempts by Loewi himself. The success of the experiment depended on a number of factors whose significance was only appreciated much later. Results varied with the season of the year, in large part due to the fact that temperature affected the speed with which Vagusstuff and Acceleransstoff were degraded.^24^ Other failures may have been due to the species of frog that was used. The frogs Loewi used contained eserine, which inhibits cholinesterase. Uninhibited, cholinesterase rapidly hydrolyses esters such as acetylcholine. Other frog species lack eserine.

Over the ensuing years, numerous successful replications of Loewi’s results, including one by Loewi at the International Physiology Congress in Stockholm in 1926, convinced many that transmission between nerve and cardiac muscle was chemical. This, however, left the identity of Vagusstuff and Acceleransstoff unknown. Loewi and others suspected that Vagusstuff might be acetylcholine,^25^ but there was still no compelling evidence that acetylcholine occurred in animals. Dale, who had earlier raised this as a concern, turned his attention instead to histamine and, in several investigations beginning in 1927, established that histamine did occur in animals. Finally, Dale and Dudley (1929) found acetylcholine as well as histamine in ox and horse spleens. Although they only found it in specific organs of two species of animals, that sufficed to overcome Dale’s earlier reason not to consider it.

Dale now began to investigate more fully whether acetylcholine was released by parasympathetic neurons when they inhibited contraction of heart muscles as well as various smooth muscles. To show this, Dale required an assay for acetylcholine at nerve terminals. Working in Germany, Feldberg had developed such an assay—a leech muscle in a bath with eserine to which blood from veins near the nerve under study could be added. The leech muscle is extremely responsive to low concentrations of acetylcholine and its contractions could easily be measured. Feldberg escaped Germany in 1933 and was recruited by Dale to join his laboratory. Together they examined chemicals extracted after stimulation of the stomach (Dale & Feldberg, 1934b) and the sweat glands in the paw of cats (Dale & Feldberg, 1934a), showing a four-fold increased response in the leech after stimulation. Based on these results, Dale and Feldberg (1934b) concluded “There is no reason to doubt that it is acetylcholine.”

Establishing that noradrenaline was Acceleranstoff resulted from a different line of inquiry. During the 1920s Cannon had been investigating sympathic stimulation. Together with Barq, he denervated the heart in cats and showed that sympathic stimulation of muscles elsewhere in the cat resulted in increased blood pressure as long as blood flow was permitted (Cannon & Bacq, 1931). They concluded that “a substance is given off from smooth muscle into the blood, that it is carried effectively by the blood to distant organs, and that it influences those organs in a favorable manner, i.e., as sympathetic impuses would influence them” (p. 411). They named the substance *sympathin*. Bacq, after returning to Belgium, carried out an analysis of the absorption spectrum of sympathin and concluded “Sympathin is surely an aromatic compound. It is probably either, like adrenaline, an amino-catechol derivative, or a mixture of amino-catechol derivatives in variable proportions” (Bacq, 1933, p. 242). The following year Bacq (1934, p. 480) proposed it might be noradrenaline, a claim that was only firmly established by von Euler (1946).

In response to these findings, Loewi (1935, p. 300) concluded his Ferrier Lecture by stating: “the ‘Vagusstoff’ is acetylcholine; I propose, therefore, to refer to it.” He remained, however, reluctant to commit to the identity of Acceransstoff with adrenaline: “In spite of all analogies, however, and although personally I am convinced of the identity, I do not feel justified as yet in assuming that the sympathetic transmitter is adrenaline, and I will therefore call it ‘the adrenaline-like substance’.” (p. 300). A year later, however, he become convinced and referred to it simply as adrenaline (Loewi, 1936).

Between them, Loewi, Dale, Barq, and Cannon provided what was regarded as compelling evidence both that the gap between activities in autonomic nerves and muscles was mediated chemically and that the chemicals involved were acetylcholine and noradrenaline. There were still important aspects of the phenomenon of chemical transmission that were not known, including both the process of transmitter synthesis and release at or near the synapse and uptake by receptors on the muscles. That is, researchers did not yet have an account of how the electrical process of transmission in neurons and muscles connects to the chemical transmission at the synapse. In this case, as in many others, establishing that a phenomenon occurs is followed by research fleshing out the account of the process. This would take time. Only with the application of the electron microscope was it possible to see the vesicles created and released at the synapse. In the meantime, researchers faced the question of whether transmission at the autonomic neuromuscular junction was exceptional or whether chemical transmission also occurred at the neuromuscular junction with striate muscles or at synapses in the central brain.

## Generalizing the chemical hypothesis to striate muscle and the central nervous system

By the time he shared the Nobel Prize for his work on chemical transmitters at the neuromuscular junction in the autonomic nervous system, Dale was already focusing more broadly—to ganglia in the autonomic nervous system and to the neuromuscular junction for skeletal muscles.^26^ In his Nobel lecture he was clear about the challenges in extending the account of chemical transmission to these cases. First, the transmission was very fast: “the phenomenon had the appearance of a direct, unbroken conduction, to ganglion cell or muscle fibre, of the same propagated wave of physico-chemical disturbance as had constituted the preganglionic or the motor nerve impulse, with only a slight, almost negligible retardation in its passage across the ganglionic synapse or the neuromuscular junction” (Dale, 1936). Proponents of the electrical hypothesis were convinced that chemical transmission would be much slower. Dale addressed this concern: the distance across the synapse or junction was sufficiently short that it resulted in minimal delay. Beyond that, Dale announced

a wider application of this conception of chemical transmission, which has resulted from researches carried out during the past three years in my own laboratory, by a number of able investigators – J.H. Gaddum, W. Feldberg, A. Vartiainen, Marthe Vogt, G.L. Brown, Z.M. Bacq. These investigations have made it possible to suggest that a fundamentally similar chemical mechanism is concerned in the transmission of excitatory effects at the synapses in all autonomic ganglia, and at the motor nerve endings in ordinary, voluntary muscle.

One of Dale’s first steps to extending the account of chemical transmission more generally was a follow up to an earlier report by Sherrington that a totally denervated muscle would contract if a nearby nerve were stimulated. Suspecting that acetylcholine might be the agent, Dale and Gaddum (1930) injected it into the vascular system and demonstrated that skeletal muscle did indeed contract. But the contractions were not comparable to those generated by stimulation of the nerve in mammals. Brown et al. (1936) addressed this objection, arguing that the differences stemmed from their inability to release acetylcholine in a sufficiently targeted fashion. They demonstrated results much more like those generated by nerve stimulation when they released acetylcholine in rapid doses that nonetheless came into contact with all fibers in the muscle. The response was still not as rapid as in response to nerve stimulation, but Brown et al. hypothesized this was due to the rapid degrading of acetylcholine by cholinesterase available locally. As indirect support for this hypothesis, they showed that moderate doses of eserine, which blocks the effects of cholinesterase, generated the expected repetitive response when a maximal stimulation was administered to a spinal cat.

Such experiments showed that skeletal muscle was responsive to acetylcholine, but not that acetylcholine was released from the nerve enervating the muscle. Dale et al. (1936) reported detecting the presence of acetylcholine upon stimulating motor nerves. To answer the objection that this could be released by the muscle itself, they showed that direct stimulation of denervated muscle did not release acetylcholine. On the other hand, when the nerve stimulated the muscle, but curare was used to prevent a response in the muscle, acetylcholine was detected.

At this point, Dale ceased to play an active role in research. His Harvey Lecture (Dale, 1937) capped this stage of his research. Dale’s collaborators, however, continued to amass further evidence for the role of chemical transmission at both junctions to striate muscles and at synapses within the central nervous system. Reviewing the work over the previous decade, Feldberg (1945) presented the case for acetylcholine in the central nervous system as “all but settled” (p. 636). He dismissed the concerns of the proponents of electrical transmission that it was too slow:

the shortness of these time intervals does not in itself provide evidence either for or against the theory that acetylcholine is the transmitter substance at central synapses. If there is sufficient other evidence available in support of the acetylcholine theory these time intervals demonstrate only (a) the rapidity with which the acetylcholine is released and acts, which must exclude any process of diffusion from the site of liberation to the site of action, and (b) the rapidity with which the acetylcholine is inactivated within the refractory period, which probably excludes any but enzymatic processes.

Feldberg did identify shortcomings of the assumption that acetylcholine was the transmitter throughout the central system. For him, though, these pointed to the possible role of chemical transmitters beyond acetylcholine: “Must we assume different transmitter substances in the central nervous system or must we assume that, in some, excitation is effected by the circulating currents from the presynaptic terminations?”

## The electricians’ resistance

Although evidence much like that amassed to support the role of chemical transmitters at junctions from autonomic nerves and muscles served to convince Dale and his collaborators that the same process figured at neuromuscular junctions to skeletal muscles and between neurons in the central nervous system, the proponents of the electrical hypothesis were far from convinced. As noted above, since they pursued their research using techniques to detect electrical transmission, the did not recognize any need to take into account anything beyond the electrical activity of neurons which they were examining. Lorente de Nó (1938), for example, maintained that the action potential in the presynaptic neuron was sufficient to generate one in the postsynaptic one, and that chemical processes were at best ancillary. Erlanger (1939) minimized the importance of the synapse as interrupting electrical transmission, comparing it to inactive stretches of nerve fibers which did not present any serious impairment to electrical transmission along the neuron. Likewise, Lorente de Nó (1939) proposed that, just as in inactive nerve segments, transmission at the synapse is due to reduced ion gradients in the vicinity of the postsynaptic neuron as a result of potassium discharge from the presynaptic neuron.^27^

**Fig. 2:**
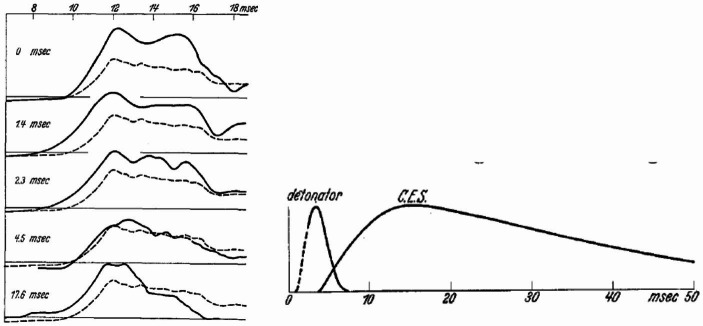
**A**. Solid lines show the increased action potentials generated when a prior stimulus is presented at the indicated intervals (generated by subtracting the action potentials produced by the first stimulus alone from that produced by the two together; the dashed line indicates the action potentials if there were no preceding stimulus). **B**. The time course of the initial detonator response and the subsequent excitatory state (labeled C.E.S. for central excitatory state). Both figures from (Eccles, 1936)

In his early work in collaboration with Sherrington, Eccles investigated the development of the central excitatory state (c.e.s., now known as the excitatory postsynaptic potential). In this work Sherrington and Eccles measured the force generated by the ipsilateral spinal flexion reflex while varying the time interval between just sub-threshold stimulation to two nerves. This allowed them to determine the time course of the c.e.s. resulting from the first sub-threshold stimulation over approximately 15 msec (Eccles & Sherrington, 1930). When Eccles turned his attention to sympathetic ganglia, he employed a variant of this approach, now measuring the number of action potentials in the ganglion. He compared the action potentials generated by stimulating a second nerve at different intervals after stimulating a first with those generated by just stimulating the second nerve. At both short (0, 1.4, and 2.3 msec) and long (17.6 msec) intervals the potential was increased by the prior stimulation, but not at an interval of 4.5 msec. (Fig. 2A). On the basis of this difference and that chemical inhibition of anticholinesterases did not affect the short interval response, Eccles identified two different phases in the response in the ganglion (shown in Fig. 2B): a fast phase that he termed the “detonator response”^28^ and a later developing slow phase that he termed the “excitatory state” (Eccles, 1937). Eccles viewed the detonator response as directly generated by the electric currents emanating from the preganglionic terminals and the later excitatory state as produced by chemical transmission: “On present evidence however, it seems that the action-current hypothesis offers a more probable explanation for direct synaptic transmission, the acetylcholine liberated in sympathetic ganglia possibly having a secondary excitatory action as already suggested.”

Eccles and O’Connor (1939) arrived at a similar account of two excitatory actions in the case of motor nerves: “(1) *The brief excitatory action by which newborn muscle impulses are set up.*. . (2) *The more prolonged excitatory action which is associated with the end-plate potential*. . Thus the evidence presented in this paper further supports the view that the same two fundamental excitatory processes are involved in all quick transmissions across junctional regions” (p. 98). In his research with O’Connor, Eccles also challenged how proponents of the chemical hypothesis interpreted the effects of eserine. Eserine had been a critical component in Loewi’s experiment demonstrating chemical transmission and had come to be understood as inhibiting cholinesterase that would otherwise hydrolyze acetylcholine. Brown et al. (1936) had shown that after application of eserine, a single nerve volley would induce repetitive discharges in the muscle fiber, which they attributed to the continuing action of acetylcholine when it was not degraded by the cholinesterase. Eccles and O’Connor failed to observe similar effects and, moreover, challenged the contention that eserine produced its effects by inhibiting cholinesterase: “we have been unable to find any unequivocal evidence that eserine action is related to the inhibition of cholinesterase with the resulting slower destruction of any acetylcholine liberated by nerve impulses at the neuro-muscular junction” (p. 38P-39P). Eccles (1937) offered an alternative explanation of eserine’s effects that didn’t face these problems—eserine made the muscle hyperexcitable by electrical stimulation.

In collaboration with Katz and Kuffler, Eccles undertook a different strategy for studying the electrical activity at the neuromuscular junction that led to him to abandon the detonator hypothesis and to seemingly become friendlier to the chemical hypothesis. Eccles et al. (1941) applied curare to block spiking in the muscle cell. This allowed them to examine the endplate potential detected as a local depolarization that resulted from nerve stimulation. They found that these potentials decremented exponentially along the muscle. However, if the potential exceeded a threshold, even with curare, a spike would be generated. Of major significance, they also observed action potentials after the effects of curare wore off. This delayed effect could not be explained by the electrical hypothesis, but it would be expected if a chemical transmitter had been protected from hydrolysis by eserine and stimulated a response once curare wore off. The following year Eccles et al. (1942) investigated the antagonistic effects of applying both eserine and curare, concluding that “by making plausible assumptions, the observed curare and eserine actions may readily be reconciled with the hypothesis that ACh, liberated by nerve impulses, is responsible for all the local potential changes at the neuromuscular junctions” (p. 228).

**Fig. 3:**
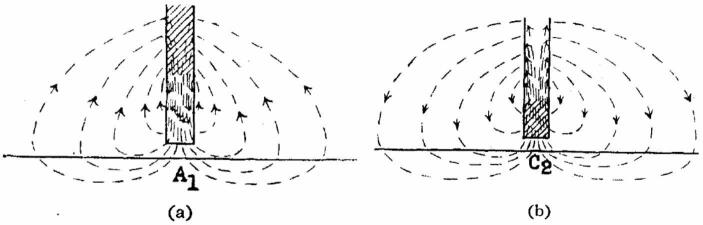
**A**. As the current moves down the axon of the presynaptic neuron (rectangle), first an anodal focus develops at A_1_. **B**. This current is then replaced with a cathodal focus that results in a current moving along the postsynaptic neuron (horizontal line). From Eccles (1946)

Within a couple years, however, Eccles made clear that he still did not embrace the chemical hypothesis. In both a preliminary report published in *Nature* (Eccles, 1945) and a more detailed report the following year, Eccles characterized the assumptions needed to render the chemical hypothesis compatible with the data ad hoc: “It is unsatisfactory that the acetylcholine hypothesis has had to be reconciled with new experimental evidence, by thus making subsidiary ad hoc hypotheses, which have not been independently testable” (Eccles, 1946).^29^ Eccles also faulted the electrical hypothesis for not making testable predictions: “The electrical hypothesis has had the grave defect that (except for the falsified isochronism theory) it has never been stated in such precise terms that it could be subjected to crucial tests.”^30^ To remedy that situation, Eccles now advanced a detailed hypothesis of how electrical currents in the nerve (perpendicular in Fig. 3) create subthreshold potentials along the muscle (horizontal): as the impulse approaches the synapse, an anodal focus will develop at the synapse with a cathodal surround (Fig. 3A) but once it reaches the terminal, the anodal and cathodal foci reverse (Fig. 3B). This then initiates a current that travels along the postsynaptic neuron. Eccles summarized his proposal:

a pre-synaptic impulse sets up electric currents exerting an initial anodal and later cathodal action on the post-synaptic membrane. The latter action, intensified by rectification, sets up a local response, which, in turn, acts as a relatively prolonged cathodal focus, from which spreads, electrotonically, the synaptic potential of the effector cell. Finally, the initiation of impulses by this synaptic potential appears to be explicable, simply, as the action of a catelectrotonus. (p. 451)

While acknowledging some things that his proposal could not yet explain, he claimed it could account for many of the features of synaptic transmission and skeletal muscle transmission. In the subsequent years Eccles set out to test various predictions from this hypothesis by recording potentials using specially designed enamel-insulated fine needle electrodes. Brooks and Eccles (1947b, p. 272) concluded “All the observations agree closely with the predictions of the electrical hypothesis of synaptic transmission.”

## The crumbling of the electrical hypothesis

**Fig. 4:**
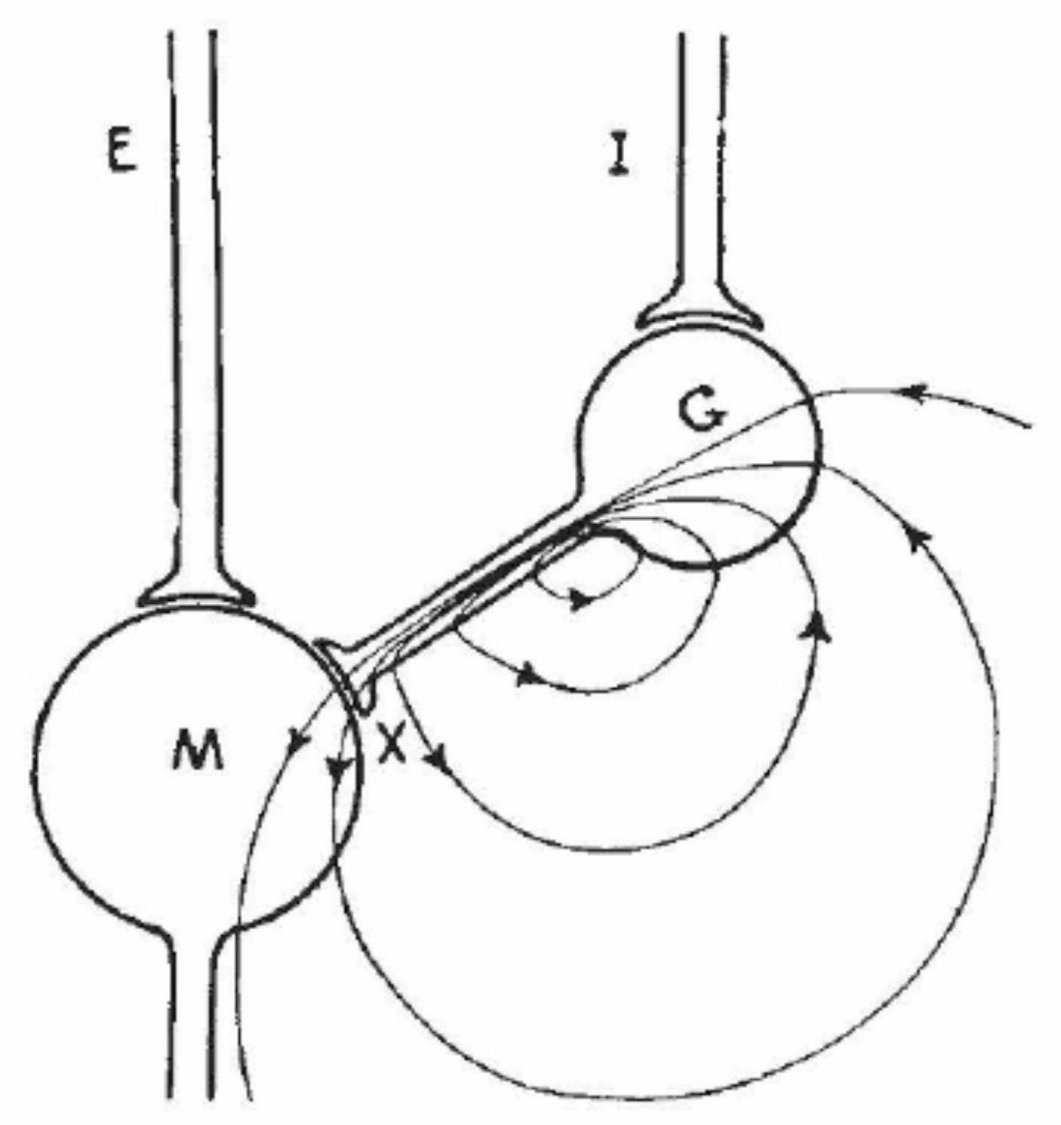
Brooks and Eccles (1947a) proposed account of inhibitory response

Eccles’ proposal in 1945 and 1946 only addressed excitatory synapses. One of the generally recognized challenges for accounts of electrical transmission was to explain inhibitory responses (something that had been the focus of Eccles early research discussed above). In the simplest form, all electrical inputs would seem to increase, not inhibit, a response, rending inhibition mysterious. To address this issue, Brooks and Eccles (1947a) advanced the hypothesis shown in Fig. 4 according to which on the pathway from the inhibitory (I) input to the motor output (M) neuron there is another neuron (G) with a short axon. Brooks and Eccles referred to this neuron as a *Golgi cell* (these cells were later renamed *Renshaw cells*) and presented them as generating anelectronic foci on the motor neurons that would block the spread of the local field responses to excitatory (E) inputs. This, they hypothesized, would render the motor neuron non-responsive to excitation.

Brooks and Eccles’ first tests of this proposal focused on the requirement that the motor neuron was in the potential field of the Golgi cell. Using an electrode positioned outside the cell, Brooks and Eccles (1948) showed this to be the case. At this time Eccles and his colleagues were developing electrodes that could penetrate neurons and recorded internal potential changes. On Brooks and Eccles’ hypothesis, such electrodes should enable them to detect a positive potential change in the motor neuron in response to stimulation by a Golgi cell. Brock et al. (1952), however, detected a result “directly opposite to that predicted by the Golgi-cell hypothesis.” They announced that Brooks and Eccles’ account of inhibition “is thereby falsified” (p. 452). The chemical hypothesis, on the other hand, made the correct prediction—with an inhibitory input the potential was expected to be more negative. Accordingly, in the discussion section they conclude “the chemical transmitter hypothesis [is] the only likely explanation.” This, they concede, “suggests further that excitatory synaptic action is also mediated by a chemical transmitter.”^31^ They do not, however, accept that the inhibitory transmitter is acetylcholine, suggesting that an unknown transmitter is involved.

Brock et al. effectively brought the war of the soups and the spark to an end, although some, including Brooks, Eccles’ co-author in proposing the electrical mechanism of inhibition, continued to hold out (see his comments in the discussion following Lorente de Nó, 1953), and Gerard (1954). Soon, however, other evidence emerged that filled out and further supported the chemical hypothesis. Using the newly introduced techniques for examining tissues with the electron microscope, Palay (1956; see also Palay & Palade, 1955) showed that the gap was smaller than suggested by light microscopy (thereby accounting for the rapid response) and revealed vesicles 200–650 Å in diameter in the presynaptic neurons. Palay proposed that these “may be considered as containing small units of a chemical transmitter, like acetylcholine.” This result was later supported by cell fractionation studies which found a fraction that separated close to that of mitochondria but contained acetylcholine (Whittaker, 1959).

## Insights into establishing scientific phenomena

Much of the literature on scientific discovery has focused on the discovery of explanations, but this relies on the prior discovery of phenomena for which explanations are offered. While there has been important philosophical analysis of what phenomena are, how they are distinguished from data (Bogen & Woodward, 1988), how they are stabilized in experimental protocols (Hacking, 1983; Feest, 2011), and how they are reconstituted (Bechtel & Richardson, 1993/2010; Bollhagen, 2021) or recharacterized (Colaço, 2018, 2020), less consideration has been devoted to how new, distinctively different, phenomena are discovered. Feest (2010, 2012) and Haueis (2023) draw attention to the importance of establishing appropriate experimental procedures and conceptual frameworks, but their focus was not on what initiated researcher’s developing new conceptual frameworks. In many cases, new phenomena first intrude on researchers. For those who recognize that something unexpected is happening, one challenge is to develop a conceptual framing of what is happening. But this is not always the pattern. In the case considered here, the phenomenon didn’t intrude. Rather, in the course of other research, some researchers identified a gap between the activities in an already recognized phenomenon (the transmission of electrical impulses along neurons and muscle cells). The challenge was to determine what filled the gap and, after soups had concluded that it was filled by chemical transmitters and receptors, to convince other researchers that there was in fact such a gap between activities that needed filling in such a manner.

For those researchers who focused on electrical transmission, the fact that the gap needed filling was concealed—they recognized synapses and neuromuscular junctions as different from neurons and muscle cells, but they recorded electrical activity before and after synapses and neuromuscular junctions and assumed they were dealing with a homogeneous type of activity (current transmission). One might expect that the gap would have presented a more pressing issue to researchers who investigated the effects of the application of chemical substances on animal tissue. They recognized that the effects were often comparable to those generated by stimulating nerves that innervated the tissue. However, except for three early investigators who each interpreted their experimental results as suggesting that a nerve elicited its effect by releasing a chemical that would then be picked up by a receptor, most of the researchers investigating the role of chemicals on tissues were not occupied with charactering how the gap between the activity of neurons was filled.

I have focused on Dale as he is particularly instructive in this respect. Early in his career he carried out some of the most detailed investigations of the effects of adrenaline, noradrenaline, acetylcholine as well as other chemicals and was clearly aware of how closely noradrenaline and acetylcholine, in particular, mimicked the effected of nerve stimulation on heart and other muscles. Yet he did not view them as actually involved in transmission between neurons and muscle. Since he was clearly aware of the claims of Langley, Elliott, and Dixon, this was not because the possibility that the gap between the electrical activity of neurons and that of muscles was filled by chemicals did not occur to him. In part, Dale’s failure to embrace their claims may be simply a reflection of his generally cautious behavior. He also had good grounds for reluctance—neither acetylcholine nor noradrenaline had yet been found in animals. On the other hand, he also did not engage in a search for such evidence. Even after the attention generated by Loewi’s experiment and the speculation that Vagusstuff was acetylcholine, he did not seek evidence that acetylcholine occurred naturally in animals and only found it in the course of investigating another chemical, histamine. The hypothesis that transmission from autonomic nerves to muscle was chemical in nature was not yet central to his endeavors.

Once Dale and Dudley (1929) did establish that acetylcholine occurs in animals, however, Dale made investigating its role at neuromuscular junctions a priority. At this point the chemical hypothesis actively framed his experimental research. For him, however, further experimental investigation was needed. It was not sufficient to show that acetylcholine is found in animals and was available to play the role identified in Loewi’s experiment. He needed to establish that it was in fact acetylcholine that was released at the neuromuscular junction. The leech assay developed by Feldman provided a means to show that acetylcholine was among the chemicals extracted from the neuromuscular junction.

The results Dale and his collaborators achieved using the leech assay, combined with Loewi’s demonstration, were sufficient to convince most proponents of both chemical and electrical transmission that transmission between autonomic nerves and muscles was chemically mediated. But ironically a crucial finding about that phenomenon—that the effect of acetylcholine on muscle developed relatively slowly—strengthened the argument of the proponents of the default electrical hypothesis with respect to skeletal neuromuscular junctions and at synapses in the central nervous system. Transmission at those locations demonstrably occurred much more quickly. At this point Dale was prepared to extend the chemical hypothesis to these contexts. His approach was not, however, designed to answer the timing question; rather, he and his collaborators sought and produced evidence that acetylcholine also occurred at these synapses and neuromuscular junctions.

Proponents of the electrical hypothesis generally did not resist the idea that the chemical hypothesis explained transmission at the neuromuscular junction of autonomic nerves and muscles but resisted its wider application. Their resistance largely took the form of arguing against the chemical hypothesis, not for the electrical hypothesis, which continued for them to provide the default assumption. Thus, they contended that the effects of eserine and curare were different than what the chemical hypothesis, as they understood it, could account for. The proponents of the chemical hypothesis did offer answers but largely went on with their own investigations. Many participants, especially on the soup side, found the controversy less engaging over time.

For Eccles, the trajectory of this research seemed to raise the electrical hypothesis from the status of a default hypothesis to one that needed to be further articulated. As he acknowledged in the mid-1940s, the electrical hypothesis had not been developed in a sufficiently specific manner to make predictions that would distinctively support it. He advanced such formulations, focusing first on excitatory connections. This proposal generated testable predictions, and they were borne out by the data he developed. Only when he advanced an electrical hypothesis for inhibitory effects, which had long been recognized as presenting a challenge for the electrical hypothesis, did he generate evidence opposite of what his hypothesis predicted.

Eccles abandonment of the electrical hypothesis was rather abrupt for someone so invested in the controversy. His proposed account of inhibition was only one possible hypothesis, but Eccles did not explore other possibilities. One explanation is that his research in the late 1930 and 1940 s had led him to recognize the strengths of the chemical hypothesis and it was not a difficult step to surrender the electrical hypothesis. The focus of the paper in which he concluded that the chemical hypothesis was correct, though, suggests a further consideration. The paper was devoted to the development of electrodes that enabled precise recording of electrical activity within neurons. Both in his hands and those of others (e.g., Hodgkin et al., 1952), these electrodes provided a means of further investigating the excitation or inhibition of postsynaptic neurons that did not depend on whether the eliciting input was electrical or chemical. Indeed, just a decade later Eccles would share the Nobel Prize for Physiology and Medicine with Hodgkin and Huxley “for their discoveries concerning the ionic mechanisms involved in excitation and inhibition in the peripheral and central portions of the nerve cell membrane.”

I conclude with several of the features of phenomenon discovery revealed in this case. Investigating a gap between activities involved in already accepted phenomena is a means to discovering that a different type of activity mediates that gap. But, as the case examined here indicates, the significance of a gap is not always obvious. Even when they recognize that there is a gap, a default assumption can allow researchers to assume that the gap is bridged without identifying a new activity. Investigators pursuing research on a different type of activity are in a better position to recognize that that new type of activity bridges the gap. But many of them may not yet be focused on the gap, at least until they establish that the entities whose activities are to fill the gap are actually present in animals. Often there are multiple gaps of the same type—in this case the gap between activities in neurons and those in cardiac or smooth muscle, the gap between activities in neurons and those in skeletal muscle, and the gap between the activities in different neurons. Determining what happens at one of them may lead some investigators to look for similar activities at other gaps while others differentiate the gaps and allow that the activity in question mediates only one type of gap. Foregoing a default assumption about the type of activity that bridges the gap may not depend on evidence for the alternative activity but require specifying the default assumption itself in sufficient detail that it makes a prediction opposite that of its competitor. It is also facilitated when the research techniques invoked to evaluate the predictions opens the path to further investigations, one that no longer depends on the default assumption.

I have only presented one instance in which researchers discovered a new phenomenon by identifying a gap between activities and determining what activity mediates between them. One case suffices to show that it is a reasonable endeavor to examine how research directed at filling gaps between activities involved in one phenomenon results in discovery of new phenomena, but only when additional examples are analyzed can we assess how generalizable are the features of this case.

## Footnotes

1 While continuing to use the term *phenomenon*, Feest (2017) challenges the juxtaposition of phenomenon with mechanisms and argues for broadening the use of *phenomenon* to encompass objects of research. In particular, she argues that in actual research, phenomena are typically “epistemologically blurry” and that much research is directed at developing “adequate descriptions of the presumed research objects.” She is correct that the term *phenomenon* has multiple uses. The notion of an object of study is an important contribution to understanding the practices of scientists, but here I am using *phenomena* for the relatively well-defined repeatable occurrences that become the targets of explanation.

2 Feest (2012) also discusses the recharacterization of phenomena in the course of research, emphasizing the important role that concept and theory construction play in the process, developing an account of operational definitions as tools employed in the process (on the role of concepts as tools, see also Feest, 2010).

3 A reviewer has objected to my use of the term *gap*, viewing it as entailing a spatial interval between the axon of one neuron and the dendrite of the next. The reviewer correctly points out that this space, the synaptic cleft, was not identified until the application of electron microscopy in the 1950s. The term *gap* need not entail a physical space but only a discontinuity between activities or processes. The discontinuity between neurons due to each having its own membrane, interrupt the transmission processes occurring within neurons and requires an account of what activity mediated between them. This indeed seems to be sense in which *gap* was used in Robinson’s (2001) title: *Mechanisms of Synaptic Transmission: Bridging the Gaps, 1880–1990*.

4 An anonymous reviewer suggests that the discovery of the synapse might be viewed as an intrusion that led to the discovery of the new phenomenon. The synapse, however, is not an instance of the phenomenon of chemical transmission intruding in the manner that electrical transmission intruded into Galvani’s experiments. It rather opened a conceptual space in which the phenomenon could later be recognized. Only with new experimental protocols was evidence for the new activity occurring across the gap advanced.

5 Valenstein (2005) adopted this characterization as the title of his very engaging historical account of the conflict. The terms *sparks* and *soups* were the actors own. Forbes (1960, p. 40), himself an active participant, writes: “For some time the controversy ran between advocates of chemical and electrical transmission at the synapse, both in the nerve centers and at the neuromuscular junction. The combatants were popularly called advocates of ‘sparks’ and ‘soup’.” (For discussion of Forbes’ own career and contributions, see Marcum, 2006.)

6 After the hypothesis that transmission between neurons involved release and response to neurotransmitters was generally accepted, researchers also identified a prominent exception. These are known as gap junctions at which ions pass directly between neurons. The gap referred to is not that between neurons but within the membranes of adjacent neurons through which the contents of one cell could enter the other. These gaps were revealed through electron microscopy of invertebrate synapses, which showed first that the dendrite of one neuron often enveloped the axon of the other (Robertson, 1953) and subsequently the aligned gaps in the neuronal membrane of adjacent neurons (Brightman & Reese, 1969; Payton et al., 1969).

7 The American researchers adopted the name *axonologists* and sponsored their own sessions at various professional meetings.

8 There were also vigorous communities pursuing such research in Germany until the 1930s and in France and Belgium. For the latter, see Bacq (1975).

9 Hacking (1983) contends that phenomena are typically only produced in the laboratory, where it is possible to achieve sufficient control to produce a phenomenon in a pure form. Even so, phenomena produced in the laboratory attract scientific interest insofar as they are taken to be indicative of what happens in the world.

10 For Schwann the main challenge was to demonstrate that the constituents of different tissues, which appeared differently in the microscope, were instances of a common type of entity, a cell. Schwann relied not just on observations but also theoretical arguments that cells were the fundamental units in which metabolic processes occur (Bechtel, 1984).

11 For a discussion of Sherrington’s role in writing this section of Foster’s textbook, see Bennett (1999, 2001). Drawing upon Sherrington’s correspondence, Tansey (1997, 2022) discusses some of the considerations that went into Sherrington’s choice of the term. Sherrington specifically opposed the term “junction” as it suggested a physical union; he preferred synapsis, viewing it as referencing the making of contact between two things that are separate.

12 Ramón y Cajal (1899, p. 1149) himself proposed an answer: “the very fact of the transmission of the wave from one neuron to another would obey the laws of chemical phenomena: in reality, the impulse provokes a chemical change in the neuronal arborizations which, working in turn as a physico-chemical stimulus on the protoplasm of other neurons, would create new currents in these. The state of consciousness would be precisely tied to these chemical changes brought about in the neurons by these nerve endings” (translation by Otis, 2001, p. 67). It is not clear what uptake this proposal had. In reflecting on his work, Ramón y Cajal (1937, p. 323) suggested a different account, appealing to electrical conduction or induction: “The continuity of substance between cell and cell being excluded, the view that the nerve impulse is transmitted by contact, as in the junctions of electric conductors, or by an induction effect, as in induction coils, becomes inescapable.”

13 Considerations of time were invoked in both directions. Cannon (1939) argued that even a few milliseconds was hard to account for on the electrical hypothesis.

14 Dale, on whom we will focus below, relates, “When I was a student of Physiology in Cambridge, in the middle 1890’s, one of our practical exercises, probably initiated by Walter Gaskell, was still to apply muscarine to the frog’s heart, and to observe the similarity of its effect to that of vagus stimulation and the annulment of both by atropine. The only explanation offered was, of course, that muscarine presumably stimulated, and atropine paralyzed, the vagus nerve endings” (Dale, 1954, p. 9).

15 Yet another line of physiological research tested the effects of chemicals released by glands in animals to determine their effects. Bayliss and Starling (1902), for example, investigated the release of secretin into the blood from what they took to be a precursor in the duodenum wall when stomach acid reached the intestine and its transport to the pancreas, where it excited excretory activity. Starling (1905) introduced the term *hormone* for such blood transported chemicals. Until fairly recently, however, hormonal transmission has been treated as a phenomenon distinct from the transmission between nerves and muscle.

16 Valenstein (2005) rejects the interpretation that Langley was proposing chemical transmission from nerve to muscle: “when Langley spoke of the ‘secretion of a special substance at the end of the nerve’ he did not mean to suggest that it was the nerves that secreted these chemical substances, for we know that he believed the substance was secreted by the muscle at the region where it was connected to the nerve.” An alternative is that Langley was proposing that the nerve released a substance, and this then combined with the receptor substance in the muscle.

17 Oliver and Schäfer interpreted their findings as showing that the adrenal glands are secretory: “like the thyroid gland, the suprarenal capsules [adrenal gland medulla] are to be regarded, although ductless, as strictly secreting glands.”

18 There was a great deal of interest in adrenaline as a pharmacological agent. After Takamine (1902) determined its composition and patented it, it was marketed by Parke, Davis, and Company as *Adrenalin*. One effect was that, to avoid patent conflicts, U.S. scientists adopted the name *epinephrine* while researchers elsewhere continued to refer to adrenaline. Use of the two names continued after researchers recognized that the biological active form was noradrenaline/norepinephrine (Davenport, 1991).

19 A comment in his Sidney Ringer Memorial Lecture indicates that Elliott had not completely abandoned the hypothesis: “The theory which I at first held to explain these facts was that the nervous stimulus consists in a liberation of adrenalin itself from the ending of the nerve on the muscle. . . I have tried in vain to discover an active substance in the muscle plates of striped muscles. . . But it is hard to forego the belief that such discoveries lie in the lap of the future” (Elliott, 1914).

20 Dixon does not specify the organism, but in his Dixon memorial lecture Dale (1935) identifies it as a dog.

21 In his obituary for Elliott, Dale (1961, p. 63) cites Elliott’s concluding line of his 1905 paper “The evidence does not conclusively disprove any of these” and responds “’Not conclusively disprove’! One might almost suppose that the transmitter theory, which has since been so well established, had then somehow got into bad company, and had only escaped conviction and sentence for want of sufficient evidence. What, then, can have produced such an apparent change in Elliott’s attitude to his own penetrating suggestion? There can be no doubt, I think, that he had been deeply discouraged by discovering that it had aroused so little serious interest among physiologists in general, and that its reception by Professor Langley, in particular, had been distinctly unfavourable.” Dale goes on to credit Elliott for his own investigations of adrenaline “I am convinced that any contribution which I may have been able to make in later years to the general development of that theme, owed its initial stimulus, and a continuing inspiration, to Elliott’s published researches and ideas, and to my personal contacts with him in those early days” (p. 64).

22 In his subsequent retelling Loewi claimed that the idea came to him in a dream that he had on two successive nights. On the first night he claims to have written down the procedure but could not interpret his notes the next day. After he had the same dream the next night, he reports going directly to the lab to conduct the experiment (Loewi, 1960). Dale (1962) reports that when Loewi first related the events to him, he didn’t go to the lab on the second night, but rather took better notes and only went to the lab the next day. Whether Loewi had such a dream is uncertain, but the idea of his experiment was not totally novel. Beyond Dixon’s earlier study, Loewi (1960) noted that in reviewing papers published from his lab he found two papers from a couple years before the experiment which utilized a similar procedure.

23 Dale reports that when he raised with Loewi why he adopted the names *Vagusstuff* and *Acceleransstoff* rather than acetylcholine and adrenaline Loewi responded that he indeed believed those were the responsible chemicals but he was being cautious.

24 Davenport (1991) states “It is important that Loewi did the experiments in February and March, for the amphibian vagus contains both inhibitory and excitatory fibers. In the winter the inhibitory fibers predominate, and electrical stimulation decreases the rate and strength of the contraction. In the summer, stimulation augments and accelerates the heart.”

25 Loewi and Navratil (1926, p. 688) did speculate “Given the extraordinarily strong effectiveness of the Vagus substance, it could even be thought that it is acetylcholine.”

26 Loewi was initially skeptical that the chemical hypothesis could be applied to the neuromuscular junction of striate muscles: “I personally do not believe in a humoral mechanism in the case of striated muscle”(Loewi, 1934). But the next year, in his Ferrier lectures, he accepted that acetylcholine was transmitted at these sites (Loewi, 1935).

27 Marcum (2006) provides a portrait of the positions electrophysiologists/axonologists took in the 1930s, especially in the U.S., highlighting the stances presented at two symposia in the late 1930s: a 1936 Cold Spring Harbor symposium on excitation phenomena (where six of the speakers addressed synaptic transmission) and a 1939 American Physiological Society symposium on the synapse. Marcum focuses on Alexander Forbes, a significant axonologist who, both in his presentations at the symposia and in several unpublished papers exhibits an ongoing struggle to accommodate both chemical and electrical components of synaptic transmission.

28 Eccles chose the name because “The discharge of an impulse is a type of explosive act of the ganglion cell, the preganglionic impulses having an action resembling that of detonator; hence the response of a ganglion cell directly related to the explosive discharge (and hence presumably responsible for the first wave of facilitation) may be called, the detonator response” (Eccles, 1936, p. 358).

29 Eccles was clearly drawing upon Popper in referring to these assumptions as ad hoc. He concludes the 1945 paper with “I wish to thank Dr. K. R. Popper for his stimulating and helpful criticism.” He also credits Popper for inspiring him to formulate the electrical hypothesis in testable terms. One of the hypotheses that Eccles singled out as ad hoc was a proposal by Dale (1937) to explain the lack of response when tissue was exposed to a bath of acetylcholine as due to prolonged exposure having a depressive effect.

30 He elaborated this criticism in 1946: “Most expressions of the electrical hypothesis of synaptic transmission. have merely stated that the electrical currents of the presynaptic impulses set up impulses in the post-synaptic cell, much as one segment of a nerve excites the next. The attempt at precise formulation by Lapicque (the isochronism hypothesis) has had to be modified so much by the recognition of the significance of additional. factors (relative durations of the pre- and post-synaptic responses, the rheobase of the post-synaptic cell), that it now states little more than the above vague formulation. Thus, the electrical hypothesis is unsatisfactory (indeed, virtually useless), because it is so vaguely expressed that it fails to give predictions that would be a fertile source of experimental tests.”

31 In contrast to the significance later attached to this paper in ending resistance to the chemical hypothesis, it is interesting to note that the result was not highlighted in the paper or its title: “The recording of potentials from motoneurones with an intracellular electrode.” The Golgi cell hypothesis is not mentioned until 19 pages into the paper.
